# Subclinical myocardial injury in patients with Facioscapulohumeral muscular dystrophy 1 and preserved ejection fraction – assessment by cardiovascular magnetic resonance

**DOI:** 10.1186/s12968-019-0537-4

**Published:** 2019-04-29

**Authors:** Edyta Blaszczyk, Ulrike Grieben, Florian von Knobelsdorff-Brenkenhoff, Peter Kellman, Luisa Schmacht, Stephanie Funk, Simone Spuler, Jeanette Schulz-Menger

**Affiliations:** 1 Working Group on Cardiovascular Magnetic Resonance, Experimental and Clinical Research Center a joint cooperation between the Charité – Universitätsmedizin Berlin, Department of Internal Medicine and Cardiology and the Max-Delbrueck Center for Molecular Medicine, and HELIOS Klinikum Berlin Buch,Department of Cardiology and Nephrology, Berlin, Germany; 2 DZHK (German Centre for Cardiovascular Research), partner site Berlin, Berlin, Germany; 3 Muscle Research Unit, Experimental and Clinical Research Center a joint cooperation between the Charité Medical Faculty and the Max-Delbrueck Center for Molecular Medicine, Berlin, Germany; 4 Department of Cardiology, Clinic Agatharied, University of Munich, Hausham, Germany; 5 National Heart, Lung and Blood Institute, National Institute of Health, Berlin, Germany

**Keywords:** Magnetic resonance imaging, FSHD, Fat, Fibrosis, Sex & gender

## Abstract

**Background:**

Facioscapulohumeral muscular dystrophy type 1 (FSHD1) is an autosomal dominant and the third most common inherited muscle disease. Cardiac involvement is currently described in several muscular dystrophies (MD), but there are conflicting reports in FSHD1. Mostly, FSHD1 is recognized as MD with infrequent cardiac involvement, but sudden cardiac deaths are reported in single cases.

The aim of this study is to investigate whether subclinical cardiac involvement in FSHD1 patients is detectable in preserved left ventricular systolic function applying cardiovascular magnetic resonance (CMR).

**Methods:**

We prospectively included patients with genetically confirmed FSHD1 (*n* = 52, 48 ± 15 years) and compared them with 29 healthy age-matched controls using a 1.5 T CMR scanner. Myocardial tissue differentiation was performed qualitatively using focal fibrosis imaging (late gadolinium enhancement (LGE)), fat imaging (multi-echo sequence for fat/water-separation) and parametric T2- and T1-mapping for quantifying inflammation and diffuse fibrosis. Extracellular volume fraction was calculated. A 12-lead electrocardiogram and 24-h Holter were performed for the assessment of MD-specific Groh-criteria and arrhythmia.

**Results:**

Focal fibrosis by LGE was present in 13 patients (25%,10 men), fat infiltration in 7 patients (13%,5 men). T2 values did not differ between FSHD1 and healthy controls. Native T1 mapping revealed significantly higher values in patients (global native myocardial T1 values basal: FSHD1: 1012 ± 26 ms vs. controls: 985 ± 28 ms, *p* < 0.01, medial FSHD1: 994 ± 37 ms vs. controls: 982 ± 28 ms, *p* = 0.028). This was also evident in regions adjacent to focal fibrosis, indicating diffuse fibrosis. Groh-criteria were positive in 1 patient. In Holter, arrhythmic events were recorded in 10/43 subjects (23%).

**Conclusions:**

Patients with FSHD1 and preserved left ventricular ejection fraction present focal and diffuse myocardial injury. Longitudinal multi-center trials are needed to define the impact of myocardial changes as well as a relation between myocardial injury and arrhythmias on long-term prognosis and therapeutic decision-making.

**Trial registration:**

ISRCTN registry with study ID ISRCTN13744381.

## Background

Facioscapulohumeral muscular dystrophy type 1 (FSHD1) is an autosomal dominant disorder and the third most common inherited muscle disease with an incidence of 1:8.000–1:20.000 [1]. Diagnosis of FSHD1 is often suspected in patients with presence of progressive asymmetric weakness of the face and shoulder muscles. However, 10–25% of patients are wheelchair-dependent [2]. Usually females have later onset and slower progression. This leads to a rate of 20% of misdiagnosed FSHD1 patients. Due to mild or unspecific symptoms females, as asymptomatic carriers, are often not or identified late [3]. The molecular test used for FSHD1 diagnosis is primarily based on the initial observation, that 95% of FSHD1 patients carry a reduction of integral numbers of D4Z4 repeats at 4q35 of the subtelomeric region of chromosome 4 [4].

Cardiac involvement in patients with other neuromuscular diseases (NMD) is more common. It may occur in up to 90% of patients in the general muscular dystrophy (MD) population, leading to heart failure and sudden cardiac death (SCD) [5]. Arrhythmias, as well as myocardial fibrosis, cause earlier clinical impairment in NMD than in other cardiomyopathies [6]. Recently, focal fat as well as focal and diffuse myocardial fibrosis could be detected in patients with myotonic dystrophy type 2 (DM2) and preserved left ventricular (LV) ejection fraction (LVEF) applying cardiovascular magnetic resonance (CMR) [7].

FSHD1 is usually recognized as a NMD with infrequent myocardial involvement, but SCD and progressive heart failure are reported [8]. Furthermore, Trevisan et al. reported arrhythmic events in 12% of the FSHD1-patients [9]. The lack of data is also reflected in the current American Academy of Neurology FSHD Guidelines [2]. The aim of this study is to investigate whether subclinical cardiac involvement in FSHD1 patients is detectable in still preserved LV systolic function applying CMR.

## Methods

### Study population

The study was approved by the local ethic committee (EA1/169/15) and all subjects gave written informed consent.

We prospectively screened 64 patients with FSHD1 of which 58 patients could be included in the study. CMR could not be completely performed in 6 patients: 3 due to arrhythmia (2 patients were excluded due to ventricular bigeminy and one patient due to atrial fibrillation), 1 claustrophobia, 1 obesity, 1 known allergy to contrast media. Finally, 52 patients (age 48 ± 15y, 36 men) were analyzed.

The diagnosis of FSHD1 was defined as partial loss of D4Z4 macrosatellite repeats units in the subtelomeric region of chromosome 4. While patients with FSHD1 present 1–10 repeats, there are 11–110 repeats in the general population [4]. Exclusion criteria were pre-existing cardiovascular diseases, malignancies or contraindication for CMR.

Patients underwent a cardiological work-up including physical examination and echocardiography to confirm a preserved LVEF. A LVEF lower than 55% was defined as an exclusion criterion [10].

A detailed patient medical history was recorded. Laboratory hematocrit was assessed just before CMR for quantification of extracellular volume fraction (ECV). Blood pressure was taken before and after CMR. Assessment of heart rhythm abnormalities was based on a 12-lead electrocardiogram (ECG) and an ambulatory electrocardiography monitoring for 24 h (Holter-ECG). Patients were also considered at risk of sudden death according to the Groh-criteria [11]. Following the criteria, patients were ranked positive if one of the following criteria was given: no sinus rhythm, PR interval ≥ 240 ms, QRS duration≥120 ms, second- or third-degree atrioventricular block. Screening for conduction abnormalities and arrhythmias was performed using a Holter. Low atrial rhythm is defined as inverted P waves in lead II, III, and aVF.

The group of patients was compared to 29 healthy subjects. The healthy population has been previously published [7, 12]. They underwent CMR and ECG. The protocol was exactly the same for both groups. Exclusion criteria were similar to the patient group. Details are given in Table 1.

**Table 1:** Patient characteristic. BP = blood pressure; HR = heart rate; BMI = body mass index; MI = myocardial infarction; * *p* < 0.05 (healthy compare to FSHD)

	All FSHD1 patients (n = 52)	Healthy subjects (*n* = 29)	FSHD1 with focal fibrosis (*n* = 13)	FSHD1 without focal fibrosis (*n* = 39)
General characteristics				
Age (years)	48 ± 15	44 ± 14	55 ± 14	46 ± 14
HR/min	75 ± 12	74 ± 10	73 ± 10	75 ± 13
Systolic BP (mmHg)	131 ± 7	130 ± 20	133 ± 6	130 ± 8
Diastolic BP (mmHg)	79 ± 8	76 ± 9	82 ± 8	78 ± 9
BMI (kg/m^2^)	25 ± 5	24 ± 3	27 ± 3 *	24 ± 5
Further characteristics				
Hypertension (%)	21.2	None	46.2	12.8
Type 2 diabetes mellitus (%)	1.9	None	None	2.6
Smoker (%)	7.7	14.8	23.1	2.6
History of MI (%)	None	None	None	None

### Cardiovascular magnetic resonance

#### CMR protocol

**Fig. 1:**
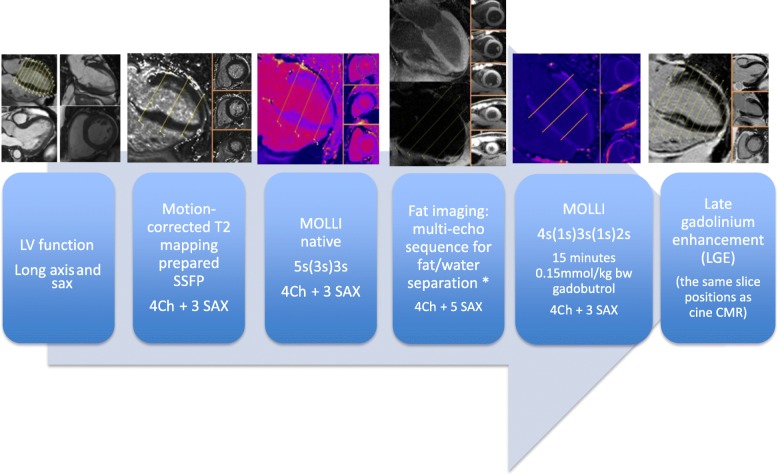
Scan protocol. LV = left ventricle; LAX = long axis; SAX = short axis; 4Ch = four chamber view; SSFP = steady state free precession; MOLLI = modified look-locker inversion-recovery; CMR = cardiovascular magnetic resonance

We applied CMR on a 1.5 T CMR Scanner (MAGNETOM AvantoFit®, Siemens Healthineers, Erlangen, Germany) using a 32-channel surface coil. The scan protocol, as recently published [7], is given in Fig. 1. Cine imaging was performed applying a balanced steady state precession sequence (bSSFP) to determine the global cardiac performance. We acquired a four (4Ch), three (3Ch) and two (2Ch) chamber view (echo time (TE) 1.2 ms; repetition time (TR), 35 ms; voxel size 1.8 × 1.8 × 6.0 mm^3^) as well as a short axis (SAX) package (TE 1.1 ms; repetition time, 63 ms; voxel size 2.0 × 1.4 × 7.0 mm^3^) to cover the LV. Short axis stacks were planned and acquired always in the same ways.

For myocardial tissue differentiation, parametric T1- and T2-mapping, fat/water-separated imaging and focal fibrosis imaging (late gadolinium enhancement, LGE) were acquired. T2- and T1-mapping were performed in basal, medial and apical slices as described recently [7, 12].

In short, motion-corrected T2-mapping was based on bSSFP gradient echo techniques (three single-shot images with T2 evolution values of 0/24/55 ms, slice thickness 6 mm) [13].

Motion-corrected T1-mapping, based on modified look-locker inversion-recovery (MOLLI) [14], was performed to detect diffuse fibrosis. A sampling protocol with reduced sensitivity to heart rate was applied [15] before and 15 min after contrast media (0.15 mmol/kg body weight Gadoteriol) application (T1 native: 5 s(3 s)3 s; T1 post-contrast: 4 s(1 s)3 s(1 s)2 s; TE 1.1 ms and slice thickness 6 mm).

A multi-echo sequence was used for fat/water-separation [16] to detect myocardial fat deposits in 4CV and five short axis. Slice position was similar to the T1 and T2 maps. Gradient echo sequence (GRE), double inversion recovery dark blood preparation, four echoes with monopolar readout, TR 450 ms, TE 1.6 ms, sth 6 mm.

Focal fibrosis imaging using LGE was performed in the same slice position as the cine imaging in 4CV, 3CV, 2CV and short axis (gradient echo sequence, breath-held segmented protocol with 10 ms echo spacing, TE of 5.2 ms, and slice thickness of 7 mm) 10–15 min after administration of contrast media. The protocol for the healthy control group was similar.

### Data analysis

Image analysis was performed using cvi^42^ version 5.3.2 (Circle Cardiovascular Imaging, Calgary, Canada).

Short axis cine images were used to determine LV volumes, mass and function by drawing endo- and epicardial contours (papillary muscles as part of the mass) at the end of the systolic and diastolic phases [17].

Both, the values of T2 and T1 maps were quantified as previously reported [7]. The qualitative survey implied the exclusion of segments in case of artifacts (e.g., caused by susceptibility effects or unintended thoracic motion) or wrong motion correction as described recently [7, 12]. In addition, we calculated the ECV by means of native and post-contrast T1 values and the hematocrit as published [18]. The hematocrit was assessed just before CMR, when the patient was already in the scanner (about 5 min before scan was started).

The quantitative analysis of mapping was performed as average value for slice and for each segment separately. The ROI was defined by the delineation of the endocardial and epicardial border of the myocardium. To ensure that blood or extramyocardial tissue are not included, safety rim of 5% was used. The segments were defined following the American Heart Association (AHA) segment model [19]. The visual evaluation of the LGE images was performed by two independent, experienced readers (SCMR Level III) to assess the presence, number and location of focal scar.

The detection of tiny fatty spots was possible applying fat/water separated imaging. Imaging was analyzed using pre-defined criteria [20]. A suspected region was considered positive if the intramyocardial fat could be either assured coexistent in the fat-separated image (hyperintense) and in the water-separated image (hypointense) or detected in one of the separated images and in the cine images and the LGE. That approach was verified by two experienced readers as recently published [7].

### Statistical analysis

All results are shown as mean ± standard deviation. The statistical analysis was performed using SPSS® Statistics 23 (International Business Machines Corp., Armonk, New York, USA). Normal distribution was analyzed graphically and with Kolmogorov-Smirnov test. For the comparisons of healthy volunteers to patients as well as within the FSHD1 group, we used an unpaired t-test and Mann–Whitney U test, respectively. A *p* value < 0.05 was considered to indicate a statistically significant difference. Correlation analyses were performed using the Spearman rank correlation coefficients. For intra- and interobserver reproducibility. Images were analyzed twice by blinded readers.

## Results

### CMR analysis

#### LV chamber quantification

No significant differences were found between patients and healthy subjects in LVEF (*p* = 0.253), LV mass Index (*p* = 0.211) as well as LV end-diastolic volume index (LVEDVi) (*p* = 0.192). (Table 2).

**Table 2:** Left ventricle characteristic

	All FSHD1 patients (n = 52)	Healthy subjects (n = 29)	FSHD1 with focal fibrosis (n = 13)	FSHD1 without focal fibrosis (n = 39)
EF (%)	63 ± 5	64 ± 4	62 ± 4	63 ± 5
EDV (mL)	126 ± 21	120 ± 22	127 ± 29	125 ± 17
ESV (mL)	48 ± 11	43 ± 11	50 ± 13	48 ± 11
SV (mL)	78 ± 15	77 ± 13	77 ± 20	78 ± 12
EDVi (mL/cm)	0.69 ± 0.10	0.73 ± 0.13	0.69 ± 0.13	0.69 ± 0.09
LVMi (g/cm)	0.55 ± 0.12	0.58 ± 0.08	0.61 ± 0.13*	0.53 ± 0.11

EF = ejection fraction; EDV = end-diastolic volume; ESV = end-systolic volume; SV = stroke volume; EDVi = end-diastolic volume index; LVMi = left ventricular mass index. * *p* < 0.05 (healthy subjects compared to FSHD1)

### Myocardial tissue differentiation

#### Focal fibrosis

**Fig. 2:**
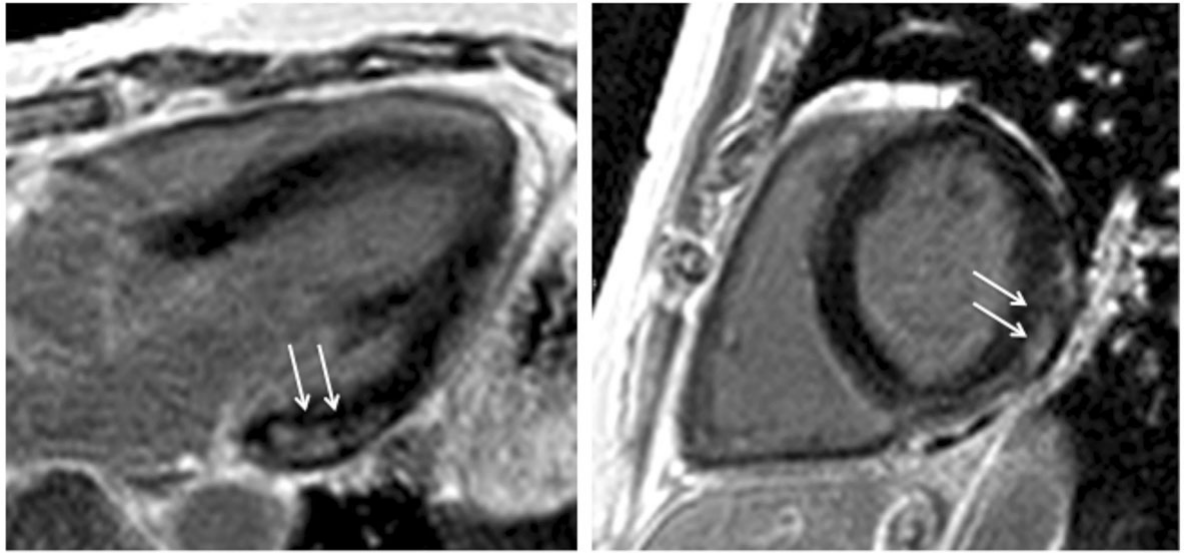
Focal myocardial fibrosis in a FSHD1 patient detected by late gadolinium enhancement (LGE) imaging. Basal inferolateral fibrosis (arrows) with non-ischemic pattern in (left) three-chamber-view and (right) short axis slice

**Fig. 3:**
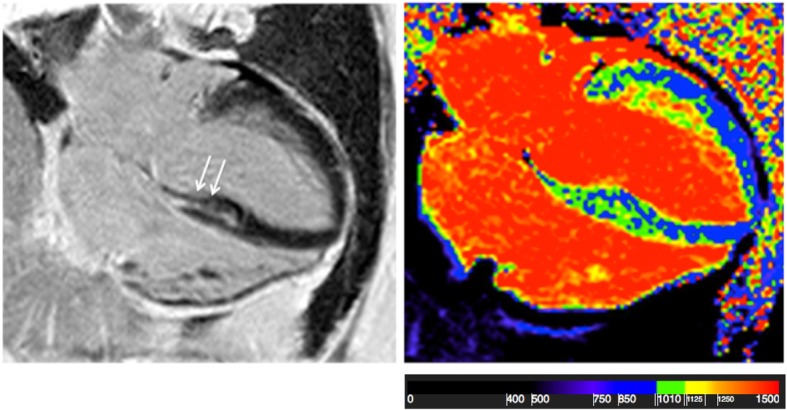
4-chamber view of an FSHD1 patient. Myocardial fibrosis in the septal wall (left) and increased T1-values (1115 ms) (right)

In the FSHD1 group, focal fibrosis was present in 13/52 patients (25%, 10 men). The three most frequent locations were the basal segments: inferolateral (Fig. 2), inferior and in the interventricular septum (Fig. 3). Only in one male patient the extension was larger and reached the mid-ventricular region. The pattern of the fibrosis was non-ischemic and mostly located intramural (10 patients, 77%), in 3 (23%) also subepicardial. Healthy subjects did not show focal fibrosis. Both readers had similar results.

There was no difference between LGE-positive and LGE-negative patients with regard to age (55 ± 14 vs. 46 ± 14 years, *p* = 0.063), LVEF (62 ± 4% vs. 63 ± 5%, *p* = 0.626) or LVEDVi (0.69 ± 0.13 ml/cm vs. 0.69 ± 0.09 ml/cm *p* = 0.991) (Tables 1 and 2).

### Focal fat

**Fig. 4:**
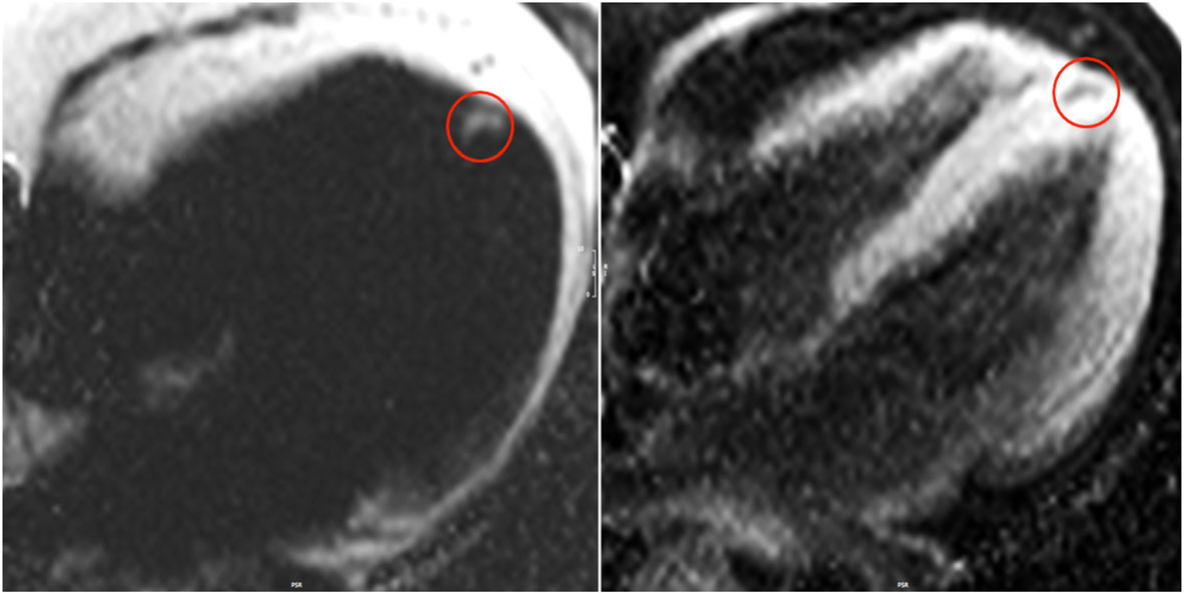
4-chamber view showing fat/water separated imaging in a patient with FSHD1. Fat accumulation detected in the apical part of the interventricular septum (red circles) presented hyperintense in the fat-separated (left) and hypointens in the water-separated image (right)

Focal fat infiltration was observed in 7/52 patients (13%, 5 men), always located in the apical part of the interventricular septal wall (Fig. 4), whereas focal fibrosis was never shown in this region. None of the healthy subjects presented fat infiltration. Three FSHD1 patients had evidence of both focal fibrosis and fat infiltration.

### Parametric mapping

The medial and basal slices were analyzed. Reliability was high for both inter- and intra-observer evaluations (r = 0.93 and 0.94, ICC = 0.82 and 0.92). Apical segments were excluded due to the high exclusion rate (in 62% artifacts and/or inaccurate motion-correction).

### T2-mapping

Basal and medial T2 maps of 52 patients were evaluated. 6/624 segments had to be excluded due to artifacts. T2 values did not differ between FSHD1 patients and the control group (basal: FSHD1 50.0 ± 3.5 ms vs. controls 49.5 ± 2.2 ms, *p* = 0.159; medial: FSHD1 51.0 ± 2.8 ms vs. controls 49.8 ± 2.4 ms, *p* = 0.108).

T2 values did not differ between LGE-positive and LGE-negative FSHD1 patients (basal: LGE(+) 50.1 ± 2.2 ms vs. LGE(−) 50.0 ± 3.9 ms, *p* = 0.932; medial: LGE(+) 50.4. ± 3.1 ms vs. LGE(−) 51.2 ± 2.8 ms, *p* = 0.391). There were also no gender differences within the FSHD1 group (basal: FSHD1 men:49.7 ± 3.7 vs. women:50.7 ± 2.9 ms, *p* = 0.058; medial: FSHD1 men:50.4 ± 2.6 vs. women:52.0 ± 2.7 ms, *p* = 0.054).

### Native T1-mapping and ECV

**Fig. 5:**
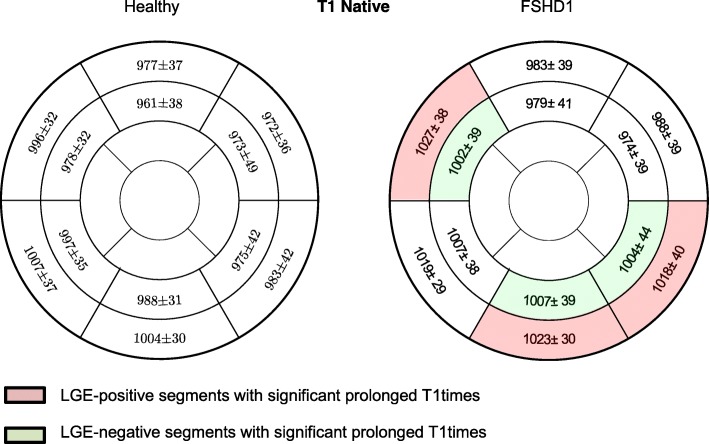
Assessment of myocardial fibrosis- Comparison of all patients with FSHD1 and healthy subjects AHA segments showing native T1 values in ms. Significant differences between healthy controls and patients were found not only in LGE-positive segments (basal inferolateral: *p* < 0.01, basal inferior: *p* = 0.033, basal anteroseptal: p < 0.01), but also within the adjacent regions (medial inferolateral: *p* = 0.032, medial inferior: *p* = 0.041, medial anteroseptal: p < 0.01)

**Fig. 6:**
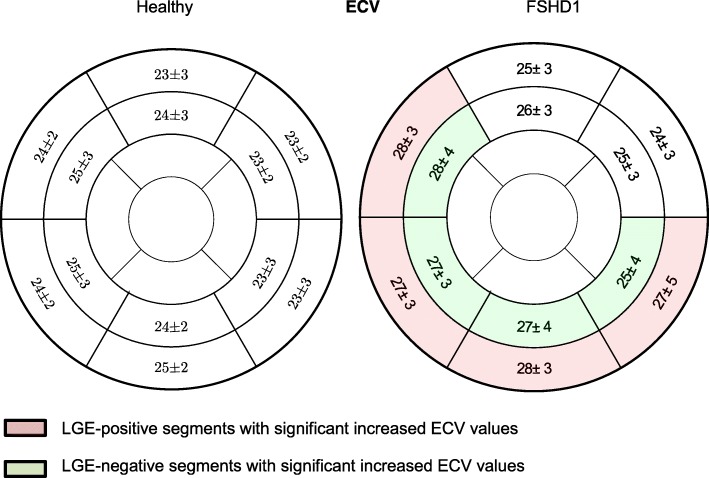
Assessment of myocardial fibrosis- Comparison of all patients with FSHD1 and healthy subjects with AHA segments showing ECV values in %. Significant differences between healthy suybjects and FSHD1 patients were found not only in LGE-positive segments (basal inferolateral: p < 0.01, basal inferior: p < 0.01, basal anteroseptal: p < 0.01, basal inferoseptal: *p* = 0.027), but also within the adjacent regions (medial inferolateral: *p* = 0.048, medial inferior: *p* = 0.021, medial anteroseptal: p < 0.01, medial inferoseptal: *p* = 0.024)

Basal and medial native T1 maps of 52 patients were evaluated. 56/624 segments had to be excluded due to artifacts and incorrect motion correction. Global native myocardial T1 values were significantly longer in FSHD1 patients compared to healthy subjects, both in the basal (FSHD1:1012 ± 26 ms vs. healthy:985 ± 28 ms, *p* < 0.01) and the medial slices (FSHD1:994 ± 37 vs. controls:982 ± 28 ms, *p* = 0.028). Compared to healthy subjects, native myocardial T1 values were longer in segments with focal fibrosis (basal inferolateral: *p* < 0.01, basal inferior: *p* = 0.033, basal anteroseptal: p < 0.01). Interestingly, Native myocardial T1 values were also increased within the adjacent medial segments (inferolateral: *p* = 0.032, inferior: *p* = 0.041, anteroseptal: p < 0.01) (Fig. 5). The same could be observed for ECV. ECV of the basal and medial slices was higher in patients than healthy subjects (basal: FSHD1: 26.3 ± 2.9% vs. healthy: 23.7 ± 2.2%, p < 0.01, medial: FSHD1:26.4 ± 3.1% vs. controls:24.2 ± 2.4%, p < 0.01). ECV was higher not only in segments with focal fibrosis (basal inferolateral: p < 0.01, basal inferior: p < 0.01, basal anteroseptal: p < 0.01, basal inferoseptal: *p* = 0.027), but also within the adjacent regions (medial inferolateral: *p* = 0.048, medial inferior: *p* = 0.021, medial anteroseptal: p < 0.01, medial inferoseptal: *p* = 0.024) (Fig. 6).

### Sex and gender differences

#### Healthy women and healthy men

There was no difference in global native T1 in the healthy control group between men and women. Native T1 in the basal slice: 978 ± 35 vs. 992 ± 18 ms (*p* = 0.275). Medial: 979 ± 33 vs. 985 ± 23 ms, (*p* = 0.394). Global ECV was also similar: Basal (men: 23 ± 2.1% vs. women:25 ± 3.2% (*p* = 0.519)) and medial (men:24 ± 2% vs. women:25 ± 3.8% (*p* = 0.866)).

#### FSHD1 men and FSHD1 women versus healthy controls

**Fig. 7:**
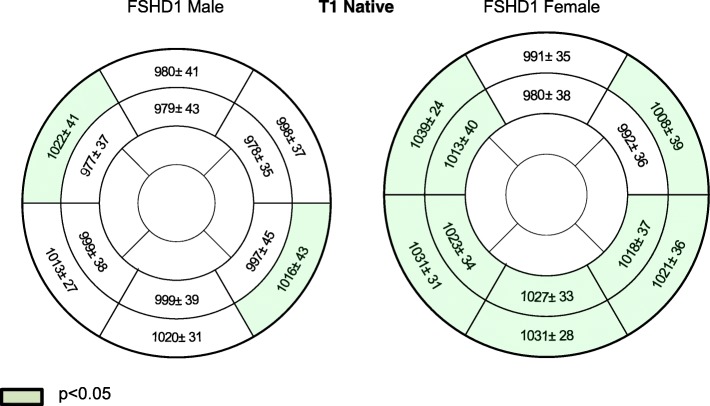
Sex differences regarding native T1 values in ms between FSHD1 patients and healthy subjects

**Fig. 8:**
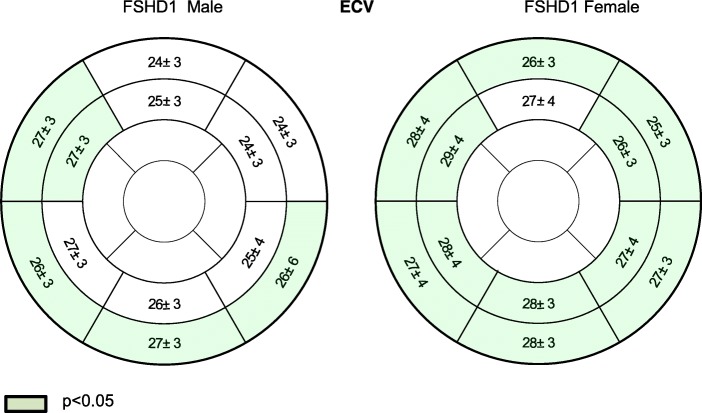
Sex differences regarding ECV in % between FSHD1 patients and healthy subjects

We compared all healthy subjects with FSHD1 men and FSHD1 women separately. In male patients, T1 native and ECV values were only significantly higher in segments with focal fibrosis. In female FSHD1 patients, T1 native and ECV values were also significantly higher in other regions. Details are given in Figs. 7 and 8.

#### FSHD1 women versus FSHD1 men

The subtle effect of sex remained significant within the FSHD1 patient group. To avoid the impact of focal fibrosis on the results, we excluded basal segments (the most common localization of focal fibrosis) from our calculations and compared T1 values and ECV in the medial slice. Both, the medial T1 values (FSHD1 men: 992 ± 26 vs. women: 998 ± 54 ms, *p* = 0.047) and the medial ECV (FSHD1 men: 26 ± 3 vs. women: 28 ± 3 ms, *p* = 0.041) were slightly increased in females.

#### Heart rhythm abnormalities

Interpretable ECGs were available in 45 patients (88%), Holter in 43 (83%). Holter was not available in 3/17 patients with focal myocardial injury. Arrhythmic events were recorded in 10/43 (23%) FSHD1 patients. Non-sustained ventricular tachycardia was detected in one patient (9 monomorphic QRS complexes, 140 bpm), premature ventricular beats > 1000 in 3/43, runs of supraventricular tachycardia (SVT) in 4/43 (124/130/140/163 bpm increasingly, the longest 146 s) and intermittent low atrial rhythm in three patients. Ventricular arrhythmias were detectable in 4/7 patients (57%) with fat deposits and in 1/13 with focal fibrosis. SVT was evident in one patient with fat deposits and in one with focal fibrosis. The patient with non-sustained ventricular tachycardia had evidence of focal fibrosis and fat infiltration. There was only one patient with positive Groh-criteria (low atrial rhythm) and normal CMR. T wave abnormalities could not be identified in any patient.

## Discussion

In our FSHD1 population with preserved LVEF, we found myocardial tissue changes such as, focal fibrosis/LGE in 25% and focal fat infiltration in 13%. Diffuse subclinical fibrosis was detectable in segments adjacent to focal fibrosis probably indicating an ongoing process. Females seem to have slightly more diffused myocardial changes. Age was not different in patients with and without fibrosis. Heart rhythm abnormalities were detected also in patients with myocardial injury.

Although we had excluded all patients with impaired LV systolic function, cardiac involvement in FSHD1 can be detected. Our findings support the case reports [8, 9, 21] describing cardiac events in this disease. To the best of our knowledge, this is the first clinical study showing the presence of myocardial changes in patients with FSHD1 with preserved LVEF in vivo.

Focal fibrosis was predominantly located in the inferolateral region. This localization is not pathognomonic for NMD as it is also described in other cardiac diseases such as inflammatory disease, mitochondrial myopathy, MD 2 or Eosinophilic Granulomatosis with Polyangiitis [7, 22–24]. Focal myocardial fat infiltration in MD was already described in case reports, but not systematically studied. Schmacht at al. showed that fat deposits are detectable in 21% of patients with DM2, interestingly only in female [7, 25].

Cardiac involvement in MD is well recognized in Duchenne muscle dystrophy (DMD) and Becker muscle dystrophy (BMD) and other muscular dystrophies with lamin A or C mutations. They are often associated with dilated cardiomyopathy and ventricular arrhythmias [26–28]. Arrhythmias and heart failure are responsible for the high mortality. This underlines the impact of an early diagnosis, as shown by Yilmaz et al. using CMR [23]. Recently, we could show that in DM2 myocardial injury is detectable already in preserved LVEF [7].

Whereas the occurrence of arrhythmias and conduction abnormalities is well known in the case of dilated cardiomyopathy [29], the association of arrhythmias and myocardial injury in patients with still preserved systolic LV-function remains unknown.

Patients with different types of MD are known to suffer from supraventricular- and ventricular arrhythmias as well as high-degree conduction disturbances like atrioventricular blocks [30]. Asymptomatic patients with myotonic dystrophy type 1 presenting the Groh-criteria were at higher risk of sudden death compared to those with normal ECGs [11]. Currently, the implication for potential device treatments in patients with MD, like the early implantation of a cardioverter-defibrillator (ICD) or a pacemaker (PM), is not obvious. The European Society of Cardiology Guidelines indicates that in this patient group the devices should be considered earlier than in other cardiac disorders [31]. Nevertheless, due to young age and clinical presentation in that population, the decision is often difficult. Additional information about myocardial tissue changes could be helpful to guide therapeutically decision making in some cases.

Myocardial fat infiltration is less studied, but due to recent technical developments the identification of small changes is less challenging [16, 20]. Pouliopoulos et al. published that fatty metaplasia in myocardial infarction is related to arrhythmia [32]. Furthermore, Lu at al. reported common prevalence of myocardial fat in dilated cardiomyopathy (DCM) patients and its significant relation to LV global function as well as possible influence on the prognosis of DCM [33]. The results of a FSHD multicenter study indicate the susceptibility to supraventricular arrhythmia as a possible feature of FSHD [9]. In our study we have registered ventricular arrhythmias in a few patients, however due to the relatively small number of participants there was no statistically significant relation between focal myocardial changes and heart rhythm disturbances. The registered rhythm disturbances do not show evidence of an increased risk of sudden cardiac death. Further studies in larger series of patients have to be performed to investigate potential relation between arrhythmias and myocardial injury in FSHD1 patients.

Parametric mapping is an innovative and reproducible method and brings unique quantitative diagnostic information about the myocardium. In the future, it may allow avoidance of contrast-media. In our study focal fibrosis as well as adjacent subtle diffuse fibrosis were detectable in a quarter of patients, using parametric mapping. Interestingly, we were able to identify differences between patients and healthy controls, but the absolute values in patients were still within the normal range. Our findings are underlining two aspects, first – quantitative mapping needs standardization and second it offers the chance to detect myocardial injury in a very early stage. Similar to our findings, Florian et al. showed in patients with different types of MD that ECV was able to detect subtle diffuse fibrosis in myocardial areas without focal fibrosis [34]. Similar to our study, CMR identified diffuse myocardial changes in DMD without the presence of focal fibrosis [35].

Sex and gender differences in different cardiac diseases are well known [36]. It has also been proven that sex has a significant influence on the development of autoimmune diseases as well as on the regulation of fibrosis and inflammation in LV muscle remodeling [37, 38]. In different MD sex and gender differences are related to cardiac manifestations, progression and outcome [39]. While female carriers of DMD rarely present clinical symptoms, cardiac involvement may develop in up to 50% of cases. In contrast to DMD males, female carriers may not clinically develop peripheral muscular disease but can present a wide range of cardiac incidents including heart failure and SCD [40].

In our study we could detect subtle sex and gender related differences in myocardial structure in FSHD1 patients compared to healthy subjects. These findings need further evaluation and confirmation. Interestingly, similar findings are described in other secondary cardiomyopathies. Cocker et al. identified in patients with viral myocarditis that males had a higher incidence of focal fibrosis, while women showed more often diffuse injury [41]. The potential impact of the significant, but small differences in T1-mapping will be a matter of further research.

Although cardiac abnormalities in FSHD1 patients are less recognized than in other MD, autopsy data reported myocardial fibrosis and focal fatty infiltrations. CMR allows the detection of myocardial tissue damage such as different types of fibrosis and fat already in the absence of functional abnormalities. Our findings in FSHD1 patients with preserved LVEF have to be elucidated in long-term follow-up studies. But it adds knowledge regarding the capability of CMR to detect myocardial injury in preserved LVEF.

### Limitations

This is a single center cross-sectional study and the sample size of the matched groups is relatively small, but all of the subjects have a genetically confirmed diagnosis and all patients are well characterized in our department of neurology. In the view of the prevalence of the disease, it should be considered as representative.

ECG as well as Holter-ECG was not available in all patients due to hyperaesthesia or logistic patient related reasons. Patients with arrhythmias that could influence the CMR scan quality were excluded from the study.

The potential impact of the significant, but small differences in T1-mapping related values have to be elucidated in further long-term follow-up trials.

We could not identify a relation between arrhythmia and native T1 mapping. But we have investigated only patients with preserved LVEF. One could expect that a larger sample size and an inclusion of all FSHD1 patients would lead to different results.

The capability of prolonged monitoring and inclusion of all FSHD1 patients to influence clinical outcomes should be evaluated in following studies.

## Conclusions

Patients with FSHD1 and preserved LVEF show focal and diffuse myocardial injury. FSHD1 men have more often focal fibrosis, while in females diffuse myocardial changes seem to be more common. Myocardial fat infiltration is detectable as well. Further longitudinal multi-center trials are needed to investigate a potential relation between heart rhythm abnormalities and myocardial injury and its impact on long-term prognosis in FSHD1.

## Abbreviations

2Ch: Two chamber
3Ch: Three chamber
4Ch: 4 chamber
AHA: American Heart Association
BMD: Becker muscle dystrophy
bSSFP: balanced steady state free precession
CMR: Cardiovascular magnetic resonance
CV: chamber view
DCM: dilated cardiomyopathy
DCM: dilated cardiomyopathy
DM2: myotonic dystrophy type 2
DMD: Duchenne muscle dystrophy
ECG: electrocardiogram
ECV: extracellular volume fraction
EDV: End-diastolic volume
EDVi: End-diasotlic volume index
EF: Ejection fraction
ESV: End systolic volume
FSHD1: Facioscapulohumeral muscular dystrophy type 1
GRE: Gradient echo sequence
Holter-ECG: ambulatory electrocardiography monitoring for 24 h
ICD: implanted cardioverter-defibrillator
LGE: Late gadolinium enhancement
LV: left ventricle/left ventricular
LVEF: left ventricular ejection fraction
MOLLI: modified Look-Locker inversion-recovery
NMD: neuromuscular diseases
PM: pacemaker
SAX: short axis
SCD: sudden cardiac death
SV: Stroke volume
TE: echo time
TR: Repetition Time
