# Correction to: Subclinical myocardial injury in patients with Facioscapulohumeral muscular dystrophy 1 and preserved ejection fraction – assessment by cardiovascular magnetic resonance

**DOI:** 10.1186/s12968-019-0541-8

**Published:** 2019-06-03

**Authors:** Edyta Blaszczyk, Ulrike Grieben, Florian von Knobelsdorff-Brenkenhoff, Peter Kellman, Luisa Schmacht, Stephanie Funk, Simone Spuler, Jeanette Schulz-Menger

**Affiliations:** 1 Working Group on Cardiovascular Magnetic Resonance, Experimental and Clinical Research Center a joint cooperation between the Charité – Universitätsmedizin Berlin, Department of Internal Medicine and Cardiology and the Max-Delbrueck Center for Molecular Medicine, and HELIOS Klinikum Berlin Buch,Department of Cardiology and Nephrology, Berlin, Germany; 2 DZHK (German Centre for Cardiovascular Research), partner site Berlin, Berlin, Germany; 3 Muscle Research Unit, Experimental and Clinical Research Center a joint cooperation between the Charité Medical Faculty and the Max-Delbrueck Center for Molecular Medicine, Berlin, Germany; 4 Department of Cardiology, Clinic Agatharied, University of Munich, Hausham, Germany; 5 National Heart, Lung and Blood Institute, National Institute of Health, Berlin, Germany

**Correction to: J Cardiovasc Magn**

**https://doi.org/10.1186/s12968-019-0537-4**

In the original version of this article [1], published on 29 April 2019, there is 1 error in the ‘Method’ section of the article.

The original publication states:

- A sampling protocol with reduced sensitivity to heart rate was applied [15] before and 15 min after contrast media (0.15 mmol/kg body weight **Gadoteriol**) application (T1 native: 5 s(3 s)3 s; T1 post-contrast: 4 s(1 s)3 s(1 s)2 s; TE 1.1 ms and slice thickness 6 mm).

However, the contrast media was **Gadobutrol**

The corrected information is also repeated below:

- A sampling protocol with reduced sensitivity to heart rate was applied [15] before and 15 min after contrast media (0.15 mmol/kg body weight **Gadobutrol**) application (T1 native: 5 s(3 s)3 s; T1 post-contrast: 4 s(1 s)3 s(1 s)2 s; TE 1.1 ms and slice thickness 6 mm).
